# Uncovering HIV Vulnerabilities Beyond the Usual Layers: Practices of Masculinity and HIV Risk Among Heterosexual Black Men in London, Ontario, Canada

**DOI:** 10.1177/2752535X251388603

**Published:** 2025-10-16

**Authors:** Roger Antabe, Godwin Arku, Erica Lawson, Josephine Wong, Winston Husbands, Isaac Luginaah

**Affiliations:** 1 Department of Health and Society, 7938 University of Toronto Scarborough, Toronto, ON, Canada; 2 Deparment of Geography and Environment, Social Science Centre, Western University, London, ON, Canada; 3 Department of Gender, Sexuality, and Women’s Studies, 6221 Western University, London, ON, Canada; 4 Faculty of Community Services, Daphne Cockwell School of Nursing, 7984 Toronto Metropolitan University, Toronto, ON, Canada; 5 Dalla Lana School of Public Health, 7938 University of Toronto, Toronto, ON, Canada

**Keywords:** African, black, heterosexual, HIV/AIDS, Ontario-Canada

## Abstract

In Canada, Black men are overburdened with HIV. Some studies have suggested that traditional masculinity practices are key drivers of new HIV infections as they endorse high-risk behaviors. However, critical scholars have also highlighted complex pathways through which Black men’s masculinity practices and their associated health impacts may be influenced by their structural circumstances in Canada. Informed by these, we draw on analyses of interviews (*n* = 13) and focus group discussions (*n* = 17) with self-identified heterosexual Black men resident in London, Ontario, to understand how they construct and practice masculinity in response to their HIV vulnerability. Using a mixed inductive-deductive thematic approach to data analysis, our findings suggest that in response to their HIV vulnerability and poor health outcomes, Black men practice progressive masculinities that empower them to engage with their health needs and help build resilience against HIV. Furthermore, race-based discrimination, stereotypes, societal expectations, and the ‘othering’ of Black men in Canada may engender the performance of traditional masculinity by some younger Black men, which may deplete their capacity to build resilience against HIV. We recommend that health policy stakeholders in Canada leverage these findings to engage Black men in addressing their structural vulnerabilities to HIV.

## Introduction

In Canada, Black people^1^ are more vulnerable to HIV. They make up less than 5% of Canada’s population, yet among new HIV cases in 2019, where race or ethnicity was known, more than 25% of the newly diagnosed identified as Black.^1^ While there is consensus that Black females are worse affected by HIV, Black males have emerged as a vulnerable group who have yet to attract policy attention in addressing their exposure to the virus.^2,3^ Specifically in Ontario, 2022 data suggest that among Blacks, males are overburdened with first-time HIV diagnoses (68%) compared to females (56%), a trend that is noted to have persisted for close to two decades.^4^ Similarly, Black males’ share of new HIV cases among all males in the province almost doubled from 12% in 2013 to 22.4% in 2022.^4^ These figures are particularly concerning as Black people constitute only 5.5% of the province’s population.^5^

While studies on men’s health increasingly point to the role of the social-cultural context on health beliefs and behaviors, there has been limited scholarship on how masculinity beliefs and practices emerge in Black men’s risk of HIV in Canada. In this paper, we sought to examine ways heterosexual Black men construct and practice their masculinity in response to their HIV risk using the context of London, Ontario, Canada. This study forms part of an Ontario-wide *weSpeak* research study that explored the HIV vulnerability and resilience trajectories of heterosexual Black men. *weSpeak* used a multidisciplinary community-based approach to engage community stakeholders, policymakers, and service providers in critical dialogue on ways to address HIV vulnerability among heterosexual Black men in Ontario and build their capacity to be resilient against HIV.

### Masculinities and Black Men’s Health

Men’s endorsement of traditional masculinities is said to negatively impact their overall health outcomes.^6,7^ Traditional masculinity may encompass perceptions, ideologies, and practices among men intended to emphasize or establish their dominant status. By this, these ideologies and practices define social interactions, sexual relations, norms on gendered roles, and expectations within society.^8^ While ultimately geared towards proving the place of men in society, traditional masculinity may be made up of two constituent parts, that is, (*i*) tied to economic achievements, including economic dominance, higher social class, and status that ensures respectability, and (*ii*) reputational dimensions which may be tied to behavioral dimensions of masculinity, including sexual prowess, physical strength and emotional suppression, risk-taking among others.^9,10^ Ideally, traditional masculinity requires a balance of these two dimensions, but it is often practiced as shaped by other dynamics such as culture, history, age, race, class, and sexuality.^11,12^

In North American societies, Black men face a daunting task in achieving parts of the ideal traditional masculinity linked to economic dominance, higher social class, and higher socioeconomic status, as these routes are often blocked to them.^12–14^ This may work to induce stress and behavioral responses with implications for their health behaviors and health outcomes.^15^ Additionally, the dynamics of the economic blockage may lead Black men to rely on the reputational dimensions of masculinity hinged on the performance of reputational behaviors which demonstrate sexual prowess, physical strength, hardiness, toughness, emotional suppression, and self-reliance, among others.^8,9,14^ This performance has adverse impacts on health behaviors such as partner concurrency, non-adherence to condom use, and poor use of preventive healthcare services^11,16^ with implications for their exposure to HIV.^8,17,18^ While ideologies of masculinity are embedded in society and practiced by many men, attempts at discussing the intersections of Black men’s masculinity with health behaviors have often been problematic.^19–21^ For instance, the interpretation of Black masculinity practices in relation to HIV risk health behaviors ignores critical issues of structural deprivation that could influence knowledge about the risk of multiple partner concurrency, the importance of adherence to condom use, and the use of HIV services.^22,23^ Additionally, it is argued that the years of ‘Jim Crow’ that legitimized the political and economic subordination of Black people in North America may have influenced legacies of their masculinity beliefs and practices and the ways they are interpreted out of context to exclude them from health interventions.^17^ Overall, it is critical to examine how Black men construct and practice their masculinities in response to their HIV vulnerability in Canada.

### Theoretical Context

This study draws on Critical Race Theory (CRT) to contextualize why heterosexual Black men are positioned to suffer poor health outcomes, including HIV infection, relative to others. CRT examines how social relations and the architecture of society, including sectors such as politics and the legal system, are grounded in racism and racial hierarchy, where racialized people experience social, economic, and political inequalities relative to the white population.^24^ Racialized populations, through covert mechanisms such as negative stereotypes and more overt discrimination like restrictions in access to resources, are confined to the margins of society.^25^ Despite the semblance of some progress in outlawing overt race-based discrimination, it has persisted, given its institutionalization in many facets of Western society. Therefore, in racially hierarchical societies such as Canada, racialized people may still face challenges in accessing resources that enhance their health.^26,27^

The years of systemic racism have contributed to health disparities, including Black men’s increased vulnerability to HIV infection. The healthcare system in North America is premised on a colonial legacy where the health needs of racialized people are neglected or not prioritized. It is noted, for instance, that the lack of cultural and structural competence in health service delivery adversely impacts the healthcare access for racialized people in Canada.^28,29^ Furthermore, a racially hierarchical society structurally predisposes Black men to live in poor housing and become underemployed or unemployed while experiencing limited health insurance coverage that works to circumscribe their access to quality healthcare.^28^

In Canada, Black men have not received the needed health policy attention and prioritization, as perceptions about their HIV risk fail to recognize it as a legitimate public health emergency needing immediate attention. This is because Black men’s disproportionate HIV disparity is stereotyped and reinforced by popular discourse as mostly behavioral and ‘self-inflicted’.^3,30^ Furthermore, the existing imagery of this population portrays them as culprits in the spread of HIV rather than people whose circumstances merit the attention of health policymakers in Canada.^25,31^ In addition, Black men’s experiences of masculinity stereotypes, including notions that ‘black heterosexual men are tough and do not show weakness’ may lead them to internalize and act out these stereotypes—further reinforcing the negative links drawn between their masculinity and HIV vulnerability.^8,32^ Furthermore, CRT is conscious of how racialized stereotypes are mainstreamed into knowledge production, and calls us to challenge the process of knowledge production, particularly in relation to race. CRT elucidates the perseverance of racialized populations in the face of social, economic, and political adversities through community mobilization, language, family, and resistance initiatives aimed at fighting inequalities.^33,34^ In line with this, CRT scholars have questioned the notions of ‘deficient’ social capital and cultural resources to fight adversity and mobilize Black communities to build their capacity to be resilient to HIV. The theory further calls for scholars in race-based research to question their positionality and assumptions about race when unpacking the disproportionate health burden of racial minorities.^35^

Furthermore, Black men’s voices in relation to their HIV vulnerability are continuously missed or misinterpreted in empirical research through the perspectives of scholars who are disconnected from their lived realities. This potentially perpetuates and reproduces stereotypes; as such, scholarships privilege the role of behavioral factors on HIV vulnerability among Black men. The present study, therefore, refocuses on prioritizing the voices of Black men as it contextualizes and documents their construction and practices of masculinities in response to their HIV vulnerability. This approach aligns with CRT’s tenet of self-representation and presentation by providing counter-narratives to mainstream discourses about the lived health experiences of Black and racialized populations.^33,36^

## Methods

### Study Sample, Data Collection, and Analysis

Data for this study form part of the qualitative phase of *weSpeak*, an Ontario-based research study focused on HIV vulnerability and resilience among heterosexual Black men in four cities: London, Ottawa, Toronto, and Windsor in 2016. This manuscript is focused on exploring how heterosexual Black men construct and practice their masculinities in response to HIV. Furthermore, the study aimed to understand how Black men’s access to resources impacted their adaptive strategies to HIV. Research Ethics was approved by the University of Western Ontario’s Non-Medical Research Ethics Board (NMREB) in 2015.

To capture the spectrum of meanings and practices of masculinity and its intersection with Black men’s HIV vulnerability, we recruited self-identified heterosexual Black men from diverse backgrounds, including different HIV serostatus, socioeconomic, geopolitical, and immigration backgrounds. As a community-based participatory study, an advisory board of eight people, comprising community members and stakeholders, was established for each study site, including London, where this study was conducted. The advisory board played a supervisory role in the recruitment process, ensuring the selection of participants was representative of the diverse spectrum of heterosexual Black men in the city. In cases of challenges with the recruitment process, such as meeting quotas for specific participant groups, the advisory board was consulted on the best approach to meeting these milestones. They also recommended the formats and dissemination channels of the research output that would be most accessible to community members. The participation criteria for the study included self-identifying as a heterosexual Black man, being at least 16 years old, being able to speak English fluently, and residing in the city of London, Ontario. Initial contacts with participants were made through flyers and posted notices in spaces where Black men frequently visit and congregate. These included Black barbering salons, churches, mosques, and some coffee shops and cafes. Other platforms for recruitment included hospitals and clinics within the city. Based on the eligibility criteria outlined earlier in this section, a total of thirty (30) participants were selected to participate in focus group discussions and in-depth interviews. Additional information on the *weSpeak* study and the recruitment strategies has been published elsewhere.^3,20,37,38^

Both Focus Group Discussions (FGDs) and In-depth Interviews (IDIs) sessions explored the meanings of acting ‘manly’ and beliefs about masculinity in relation to how participants address their health needs and practices, including HIV prevention. The impact of structural violence and poor economic opportunities on healthcare access was discussed. IDIs allowed participants to delve into sensitive discussions, including sexual practices. Furthermore, IDIs were used to protect the identity of Black men living with HIV from accidentally disclosing their HIV status. To facilitate resonance among participants, they were grouped into similar age cohorts for the FGDs.^39,40^ The FGDs were kept to a small number of participants to ensure group cohesion and encourage participation.^41,42^ In line with the study’s ethics protocol, all participants signed a consent form. The sociodemographic characteristics of the participants are provided in Table 1.

**Table 1.** Socio-Demographic Characteristics of In-Depth Interviews and Focus Group Participants.

Participants characteristics	Frequency (*n* = 30)	Percentage %
Ethno-identity		
African	12	40.0
Caribbean	10	33.3
Black	8	26.7
Region of birth		
Africa	7	23.3
Caribbean	5	16.7
Canada	16	53.3
Other	2	6.7
Immigration status		
Canadian citizen	23	76.7
Landed immigrant/Permanent citizen	4	13.3
Refugee/Protected person claimant	2	6.7
Other/Student study Visa	1	3.3
Age		
16–24	8	26.7
25–38	12	40.0
39+	10	33.3
Employment status		
Employed	12	40.0
Self-employed	6	20.0
Student/part time	7	23.3
On disability	3	10.0
Unemployed	2	6.7
Annual income (CAD$)		
<20,000	18	60.0
≥20,000	12	40.0
Educational attainment		
High school or less	12	40.0
Some college/university/completed college/university	18	60.0
Masters or higher		
Marital status		
Married or common law	10	33.3
Single (never married/divorced/separated)	20	66.7
HIV Status		
HIV+	3	10.0
Never tested/don’t know/HIV-	27	90.0
Religion		
African traditional	1	3.3
Christian	22	73.3
Muslim	3	10.0
Other/no religion	4	13.3

The IDIs and FGDs lasted between 45–60 and 60–90 minutes, respectively. Both were semi-structured. Emerging themes from FGDs, including intersections of masculinity and sexual practices, systemic barriers, and stereotypes, were further probed during IDIs. Before each session, participants’ consent was sought to audio-record. Participants could also request that the audio recorder be turned off or that the interview be discontinued at any point. Participants were told their identities would be protected through the use of pseudonyms in manuscripts and project reports. Overall, there were three focus groups, each comprising heterosexual Black men of similar age cohorts: 16-24 years (*n* = 5), 25-39 years (*n* = 5), and 39+ years (*n* = 7). This was followed by 13 IDIs, which included three HIV seropositive participants. Each participant received a $20 honorarium after the session.

To analyze the interviews, audio recordings were transcribed verbatim and exported into NVivo, a qualitative data analysis software. Grounded in CRT, we were conscious of how dominant ‘knowledge’ about Black men’s masculinity and the drawn links to poor health outcomes may re-echo long-held stereotypes — which some Black men tend to internalize.^43^ Informed by this understanding, the team, comprising four research assistants and a co-principal investigator, used a mixed inductive-deductive thematic approach to data analysis. Thus, our theoretical grounding in CRT guided the identification of parts of our themes, while the data helped identify the rest.^44,45^ Guided by a codebook developed by the entire team across all the research project sites and members of the advisory committee, team members at the London site read through the transcripts several times before identifying themes. This was subsequently followed by line-by-line coding according to our specific research objectives.^46,47^ Four members of the research team identified themes on the meanings and practices of masculinity. Coded transcripts were exchanged between research assistants to cross-check and ensure coded statements were representative of assigned codes. While there were mostly agreements among the team members on the assigned codes, the few disagreements were brought to the entire team for discussion and resolution. Themes from the coding process were shared with members of the advisory board, who provided insights on how the themes aligned with their knowledge about Black men’s masculinity beliefs and practices in the community. The co-principal investigator, who has experience in qualitative research, reviewed the transcripts and themes to ensure consistency. Finally, a researcher with extensive experience in qualitative research, who was not part of this research project, reviewed the transcripts and noted that the themes were representative of the participants’ views. These processes were embedded in the data analysis stage to prevent analytical drift and ensure rigor.

## Results

Our findings revealed varied beliefs and practices of masculinity from traditional to progressive among Black men in the study context. For instance, younger age cohorts of Black men were more inclined to internalize and subscribe to forms of traditional masculinity. Through this, they intended to attain and authenticate their identity as heterosexual Black males. This included acting out stereotypical depictions of Black men’s masculinity as sexual performance.

In contrast, as Black men age, they tend to embrace and practice more progressive forms of masculinity. Older cohorts of Black men subscribed to forms of masculinity that centered on self-care, prioritizing one’s health and well-being. Participants identified structural risk factors, including stereotypes and expectations, stigma, and limited engagement with service providers, as key drivers of HIV vulnerability in the community. The observed heterogeneity and fluidity of beliefs and practices of masculinity by Black men enabled them to engage with their health needs, including those directly related to their HIV vulnerability.

### Practicing Traditional Masculinity as Part of Perceived Black Identity

Some younger Black men linked their performance of traditional masculinities to an ‘authentic’ Black identity. To them, the performance of these masculinities defined and reinforced their identities as men, heterosexual, and Black. However, these practices mirrored entrenched stereotypes and expectations of Black masculinity. Endorsing and acting out these masculinities risked exposing, particularly younger Black men, to HIV. A participant in an IDI remarked on how Black men are expected to demonstrate their masculinity in ways that re-echo the stereotyped Black masculinity.

I think the way women view Black men also plays a role in our image. I believe women view Black men differently than they would view other men. They view Black men as more powerful and not expressing too much love or any form of weakness. I think that women look at Black men as very strong, I would say leaders, but also very powerful men in general, and it affects the way we act [FG 27 years, African Identified]

In the context of their stereotyped masculinity, Black men are not to show weakness and are expected to exhibit behaviors that reaffirm their heterosexual status. Over time, these stereotypes have been normalized, with some Black men exerting pressure on their peers to conform to these stereotypes. However, doing so has implications for building resilience against HIV.

I have been trying to practice abstinence, and if I would talk to brothers [friends] from Toronto on the phone, the number one thing they want to hear about are stories of [the number of] women I have had sex with [IDI 32 years, Black Identified]

Similarly, a participant acknowledged how the need to prove their masculinity leads them to endorse and act out masculinity as sexual performance. Gaining respectability and reputation among peers through the performance of this masculinity created a competitive urge among some Black men to have as many sexual partners as possible.

I would always compare, I would feel less than my other black friends who have two, three or four girls on the go [IDI 25 years, Caribbean Identified]

Younger Black men, in particular, felt overwhelmed by society as it pressures them to conform and enact the stereotyped masculinities imposed on them. Kwasi, a 20-year-old in a focus group, also observed this pressure when he stated, *‘...i feel like there is pressure from society for me not to be able to turn a woman down if she asks or offers me sex.”*

### Pushing Back on Black Masculinity Stereotypes

Black men acknowledged how entrenched stereotypes from long-standing perceptions about their masculinity and sexuality have become overarching, imposing expectations on them. A participant observed the difficulty in separating these stereotypes and expectations from ways they practiced their masculinities, albeit admitting this increased their exposure to HIV.

Well, [the perception that] a black guy is only concerned with sex affects our community’s HIV risk. I see in our [Black] society that these perceptions can influence people, and the more outlandish the thoughts seem to be, the more people want to go there [IDI 59 years, Black Canadian Identified]

Another participant remarked on how Black men’s masculinity and sexuality are notoriously merged in these stereotypes. That is, to be a Black man means to be heterosexual. Black men are, therefore, expected to act in ways that show that they are heterosexual. Black men, however, show awareness of this and resist the conflation of their masculinity with sexuality and the perception of their masculinity as sexual performance.

A lot of times, people don't understand that our sexuality and masculinity are two different things. We label things like sleeping with many women as masculine, and that plays into our masculinity. Sleeping with a lot of women should not make you more of a man, but it does; that is how society plays it [FG 40 years, Caribbean Identified]

Additionally, Black men’s masculinity is stereotyped to be solely centred around the showings of hardiness, toughness, and suppression of emotions. However, they were aware that this characterization of their masculinity was problematic.

As a Black man, I’m supposed to be a super masculine guy…. I'm not supposed to show much emotion whatsoever; I'm supposed to be this tough cookie, and no matter what happens, I have to be big and not cry. I can’t show emotion, and I think that is the social norm for any Black group of people [FG 20 years, African Identified]

Participants consistently acknowledged the pressure imposed on them in subscribing to traditional ideas about masculinity, such as dating multiple women, being dominant in relationships, being tough, being detached from emotions, and not showing weakness.

### Meeting Healthcare Needs as a Tenet of Black Masculinity

Findings also pointed to progressive masculinities useful in improving Black men’s overall health and reducing their HIV risk. According to Calton et al.^48^ (page 41), progressive masculinity is “a form of masculinity emphasizing movement away from traditional male gender roles that are detrimental, restrictive, and oppressing of women, and instead toward volitional and egalitarian behaviors, values, and beliefs.” Among our participants, such progressive masculinities are linked to their increased awareness of the deleterious impacts of neglecting their health needs in the performance of assigned responsibilities. A participant in an IDI emphasized the importance of self-care as a tenet of masculinity and being manly.

….being manly is to care for your health first. You can then take care of everyone else afterward. But you can't care about anybody if you don't care about yourself [IDI 57 years, Black Identified]

The finding that Black men construct manly behaviours to include taking care of their health reveals an empowering idea of progressive masculinity that enables them to engage with their healthcare needs. Participants also discussed how their understanding of behaving manly included their participation in a range of activities traditionally considered feminine. For instance, a participant observed.

Manly is providing for yourself, taking care of your health, going grocery shopping, and washing your clothes. Those are all manly things [IDI 32 years, Caribbean Identified].

Constructing masculinity to encompass previously feminized activities paves the way for addressing HIV as a lingering health issue among Black men. These changes are happening alongside increasing awareness about platforms and spaces that propagate and entrench stereotypes about Black men’s masculinity. A research participant suggested that biased and stereotypical media portrayals of Black culture underpin Black men’s deleterious masculinity practices.

Being manly is to take personal responsibility for everything in your life, rather than the propaganda in the media where being manly means you have to pack a gun, sell drugs, do violence….That is what these people in my social group are being misled to think of what is manly and what is not [IDI 25 years, Caribbean Identified]

These progressive masculinity practices help position heterosexual Black men to safeguard their health and, more specifically, build resilience against HIV.

### Vulnerability and Progressive Masculinity

Black men navigate feelings of vulnerability by confiding in friends, family, and close acquaintances. Through this, they communicate their vulnerability while demonstrating a willingness to seek help when needed.

I have been dealing with that feeling a lot lately. I have learned to be more open and communicate because talking about it [vulnerability] will make you feel a lot better in ways you wouldn't think. You would think the same problem is going to be there, but just talking about it does make a difference [IDI 26 years, Black Canadian Identified]

In addition to friends and close acquaintances, Black men also indicated seeking professional help in dealing with their vulnerability.

..having friends and professionals that I consider to be smart, intelligent, likable people, willing to talk to me, and say I am okay. And then realizing that I am okay myself [IDI 59 years, Black Canadian Identified]

Practices of progressive masculinity, which appear common among Black men, underpin their health-seeking behaviors, including their engagement with healthcare professionals. However, some participants noted that health service organizations and institutions do not focus on the specific healthcare needs of Black men. A participant living with HIV commented on the paucity of services for heterosexual Black men.

I don't see a lot of heterosexual services dealing with HIV out there. If we are going to talk about the heterosexual point of view, there isn't [IDI, 45 years, Caribbean Identified]

Despite their vulnerability to HIV, some Black men were not aware of HIV as an issue in the community. This may be particularly the case for recent immigrants who lacked access to information on the HIV epidemic within the Black community in Canada. Participants acknowledged the lack of structural competence and neglect in providing information about HIV services in their community.

Politics doesn't give us the right information that we need about HIV. …. They don’t say you guys have the highest rate of HIV, and therefore, we should find a way to address it. They don't care about that [FG Age 27, African Identified]

The preceding quote implies that Black men are open to engaging with policy stakeholders and service providers in dialogue aimed at reducing their HIV vulnerability. However, stakeholders, particularly service providers, have yet to engage them on ways services could be improved and oriented to encourage their use by Black men. The lack of dialogue may be contributing to the lack of cultural competence in HIV service delivery, as noted by some participants.

## Discussion

This study aimed to examine ways heterosexual Black men construct and practice their masculinities in response to their HIV vulnerability using the context of London, Ontario, Canada. Our findings demonstrate that beliefs and practices of masculinity linked to Black men’s HIV risk are not exhaustive of the ways masculinities are practiced among them. Guided by our theoretical insights from CRT, it emerged that participants who endorse traditional notions of masculinity may be doing so within the broader context of decades of structural violence, where Black men are expected to demonstrate these forms of masculinity to authenticate a stereotyped identity. In line with the observation by Castle, Bell, and Harris^49^ and King^50^ in the United States, and cues from CRT, we argue that perceptions about Black men not adequately responding to their HIV risk are part of the larger discourse that problematizes their masculinity practices as the bane of their poor health. Indeed, evidence of Black men embracing progressive masculinities to engage their health and ensure their well-being suggests their self-awareness about the adverse impact of traditional masculinity on their overall health experiences. This finding may also be in the wake of increasing awareness that the forms of traditional masculinities imposed on them through mediums such as the media, entertainment, and religion predispose them to poor health and increase their vulnerability to HIV.^51,52^

Our findings about these progressive masculinity practices critique and discount a homogenous Black traditional masculinity that is deleterious to Black men’s overall health and well-being.^25^ An earlier study by Ward^52^ noted that these imposed masculinities on Black men are continuously opposed and resisted by Black men, allowing them to redefine masculinity in ways that meet their best needs. Specifically, in Canada, Husbands et al.^19^ established similar findings among Black men residing in London and Toronto, Ontario, noting that they engaged their personal and interpersonal vulnerabilities in creative and strategic ways and invested emotionally and practically in their health. The progressive masculinities practiced by heterosexual Black men empower them to engage their health needs, including building resilience against HIV, as they prioritize meeting their healthcare needs and construct seeking healthcare as part of manly behaviors.

As Black men become conscious of the debilitating health impacts of living out stereotypes, they are moving away from previous masculinity expectations, which are often influenced by long-standing marginalization.^19^ Findings from this study further suggest that heterosexual Black men demonstrate their awareness of their HIV vulnerability by resorting to resources that can help them overcome this adversity. Indeed, participants may be engaging in resilience-promoting activities that improve their overall health. These include discussing feelings of vulnerability with trusted acquaintances and health professionals, despite earlier suggestions that they are notoriously secretive about their health needs. Consistent with Husbands et al.^19^ Black men show resourcefulness in building their capacity to be resilient against HIV, notwithstanding the absence of health policies targeting them.

Despite their structural vulnerabilities, our findings further demonstrate that Black men are surmounting existing barriers to promote their resilience and reduce their HIV exposure. Although they face increased barriers that potentially impact their healthcare access, Black men show commitment to practicing more progressive masculinity ideas, which offer them the opportunity to reduce their exposure to the virus. However, by not targeting Black men appropriately, health promotion policies and programs endanger Black men’s capacity for resilience. Indeed, earlier work showed that Black men’s decreased use of health services contributing to their HIV vulnerability is linked to structural factors, including limited economic engagement, racism, and microaggressions.^21,30,53^

These findings suggest the need for an equity-oriented health practice and policy to address the disproportionate burden of HIV among heterosexual Black men. It is critical to disabuse the mindset of healthcare professionals that the HIV vulnerability of heterosexual Black men is neither ‘self-inflicted’ nor due to their subscription to deleterious traditional masculinity. Addressing such stereotypes will ensure that heterosexual Black men visiting health care outlets and in their respective social interactions are not treated as ‘culprits’ of the spread of HIV, but rather, as victims who need empathy and attention in these spaces as they seek health and social services. Creating the platform for heterosexual Black men to have equitable access to HIV services is underpinned by our finding that reveals that they engage in resilient building practices against HIV. An equity-informed health policy would require leveraging on Black men’s willingness to seek critical resources to improve their health. This suggests the urgency of health policy stakeholders to partner with them to design targeted policies for reducing their HIV vulnerability. Overall, the commitment to health equity for this structurally exposed population requires a paradigm shift in the ways we conceptualize their HIV vulnerability. This also requires the initiation of specific and targeted policies, along with dedicated funding, for community engagement and building partnerships with key stakeholders. Through this, the community can have a permanent voice in the ways their health outcomes are interpreted and the policies that are designed to address their disproportionate HIV burden.

## Conclusion

This study reveals that heterosexual Black men construct and subscribe to progressive concepts of masculinity that allow them to engage with their health needs. The practice of such progressive masculinity ideologies in relation to their health generally further demonstrates Black men’s willingness to engage with their HIV vulnerability. However, as argued by Geary^25^ and Bowleg et al.,^35^ racism and structural inequalities have worsened the poor health experienced by the Black population while privileging research and discourses about a problematic Black masculinity. There is an urgent need for health policy stakeholders in Canada to engage the Black community, specifically heterosexual Black men, to frame policies and interventions that focus on reducing their exposure to HIV and vulnerability to poor health. The revelation that the meanings of masculinity among Black men are not opposed to meeting their health needs presents an opportunity for stakeholders. The community should be engaged to craft culturally relevant and structurally competent programs aimed at increasing heterosexual Black men’s use of services at ASOs. It is also critical to remove structural barriers, including anti-Black racism, microaggressions, and economic exclusion, as part of a holistic framework towards HIV prevention in London, Ontario, and similar contexts in Canada. Based on these findings, we call for a more critical approach to studying the intersections of Black masculinity and HIV vulnerability.

## Consent to participate

We obtained both verbal and written informed consent from all participants in this study. No personal identifier has been used in the quotes in this manuscript; therefore, participants remain anonymous.

## Note

1 Include Black people of African descent and the African Diaspora in Canada. Some sources capture this population as ACB (African, Caribbean, and Black).
